# Adsorptive Stripping Anodic Voltammetric Determination of Thioctic Acid in Bulk and Pharmaceutical Formulations

**Published:** 2009-03

**Authors:** Nawal A. Alarfaj

**Affiliations:** *Department of chemistry, College of Science, King Saud University (Girls), Riyadh, Saudi Arabia*

**Keywords:** thioctic acid, adsorptive anodic stripping voltammetry, pharmaceutical analysis

## Abstract

The electrochemical behavior of thioctic acid at the hanging mercury drop electrode was studied in different supporting electrolytes. In Britton - Robinson buffer pH 3.29, the square – wave adsorptive anodic stripping voltammogram of thioctic acid exhibited a single well – defined anodic peak at about -0.21 V vs Ag/AgCl. The peak response was characterized with respect to pH, preconcentration time, accumulation potential, supporting electrolytes, frequency, scan increment and pulse amplitude. Under the optimized conditions, a fully validated, simple, high sensitive, precise and inexpensive square – wave adsorptive anodic stripping voltammetric procedure was described for determination of thioctic acid in bulk form and tablets. The obtained results were in good agreement with those obtained with the reference method. The calibration graph was linear over the range 5 × 10^-8^ - 9 × 10^-7^ M (r=0.9997) with accumulation for 60 s at –0.4 V vs Ag/AgCl and the detection limit was ca. 1.2 × 10^-8^ M.

## INTRODUCTION

Thioctic acid is 1,2-dithiolane-3-pentanoic acid. It is used for treatment of liver dysfunction and diabetic neuropathy and as antidote to poisonous mushrooms [Amantia species] (1, 2).

The reported methods for the determination of the drug include spectrophotometry (3-5), spectrofluorometry (6), electro – analysis (7-14), enzyme immunoassay (15-17), capillary electrophoresis (18), liquid chromatography–mass spectroscopy (19) gas chromatography – mass spectroscopy (20) and high performance liquid chromatography (21-30).

Adsorptive stripping voltammetric analysis especially with the square – wave waveform is an extremely simple and sensitive technique that can be used for analysis of drugs without the necessity for extraction steps prior to the assay. Moreover, the square – wave voltammetry is a large – amplitude differential technique in which a waveform composed of a symmetrical square waves is applied to the working electrode (31). The current is sampled twice during each square – wave cycle, once at the end of the forward pulse and once at the end of the reverse pulse. The resulting peak current is proportional to the concentration of the analyte. Excellent sensitivity occurs from the fact that the net current is larger than either the forward and reverse components. Coupled with the effective discrimination against the charging current, very low detection limits can be attained. The major advantage of square – wave voltammetry is its great speed. The effective scan rate is given by square – wave frequency ƒ (in Hz) and the step height ΔE_s_ as ƒ ΔE_s_.

To date, thioctic acid was determined by adsorptive cathodic stripping voltammetry (14) up to 1.6 × 10^-6^ M and the detection limit was 1 × 10^-7^ M. No adsorptive anodic stripping voltammetry was reported for the assay and quantification of thioctic acid in the literature. Hence, the current electroanalytical research aimed to study the square – wave voltammetric behavior of thioctic acid and its interfacial adsorptive cathodic accumulation onto the hanging mercury drop electrode (HMDE). Based on the results obtained a simple, sensitive and low cost square wave adsorptive anodic stripping voltammetric (SW – AdASV) procedure was developed for the direct determination of thioctic acid in its pharmaceutical formulations.

## EXPERIMENTAL

### Apparatus

All SW – AdASV measurements were carried out with the Electrochemical Analyzer 746 VA Trace Analyzer, Metrohm, Herisau, Switzerland. A three – electrode system, composed of a HMDE as the working electrode, an Ag/AgCl reference electrode and a platinum wire as auxiliary electrode, was used. Stirring of the solution in the electrolysis cell was performed using a complete stirrer comprises as a part of the electrochemical analyzer to provide the convective transport during the preconcentration step. The whole measurements were semi – automated and controlled through the programming capacity of the apparatus. The data were treated through an external printer attached to the two RS 232 interfaces of the 746 VA Trace Analyzer.

A Hanna pH211, Romania pH – meter with combined glass and saturated calomel electrodes was used for the pH measurements of the supporting electrolytes.

The de-ionized water used throughout the present study was supplied from Elgastat Micromeg, England.

### Materials and reagents

Thioctic acid was provided by Eva Pharm for Pharmaceuticals & Medical Appliances, Cairo, Egypt. Phrmaceutical preparations containing the drug were obtained from commercial sources.

Britton - Robinson buffer (32) (0.08 M), pH range 2-11, Walpole acetate buffer (32), pH range 3.6–5.6, sodium sulphate (Fluka, Switzerland) 0.1 M, sodium nitrate (Fluka, Switzerland) 0.1 M and potassium chloride (Fluka, Switzerland) 0.1 M solutions were prepared by dissolving materials (analytical grade) in specific volumes of de-ionized water and were used as supporting electrolytes.

### General analytical procedure

Stock solution (1 × 10^-3^ M) of thioctic acid was prepared in methanol (BDH, Ltd, UK). This solution was further diluted with methanol to give the appropriate concentrations of working standard solutions. Aliquots of these solutions in the concentration range 5 × 10^-8^ – 9 × 10^-7^ M were transferred into a series of 25 ml volumetric flasks and diluted to the mark with Britton - Robinson buffer of pH3.29. Each solution was transferred into the electrolysis cell, then was purged with pure nitrogen. The accumulation potential of – 0.4V versus Ag/AgCl was applied to a new mercury drop while the solution was stirred at 400 rpm for 60s. At the end of the accumulation period, the stirring was stopped and a 5s rest period was allowed for the solution to become quiescent. Then the voltammogram was recorded by scanning the potenlial toward the positive direction over the range – 0.4 to + 0.2V using the SW mode. All measurements were made at room temperature.

### Analysis of tablets

An accurately weighed quantity of the mixed contents of ten pulverized tablets equivalent to 10.0 mg of the drug was transferred into a 50 ml volumetric flask. Then methanol was added to the mark. The flask with its contents were sonicated for 15 min and filtered. The desired concentrations of the drug were obtained by further dilution with methanol. An aliquot volume of this solution was transferred into a 25 volumetric flask and diluted to the mark with Britton - Robinson buffer of pH 3.29. The above general analytical procedure was followed. The nominal content of the drug was determined either from a previously plotted calibration graph or from the regression equation.

## RESULTS AND DISCUSSION

### Stripping Voltammetry

The determination of thioctic acid was done on the HMDE by SW-ASV. During a preconcentration step of 60s at -0.4V on the electrode surface, the disulfide present in the analyte is reduced to mercaptan in the adsorption process and by the transfer of two electrons, as shown in Eqs (a) and (b):

(a) $$\mathrm{RSSR}+\mathrm{Hg}\to {\left[{\left(\mathrm{RS}\right)}_{2}\mathrm{Hg}\right]}_{\left(\mathrm{ads}\right)}$$

(b) $${\left[{\left(\mathrm{RS}\right)}_{2}\mathrm{Hg}\right]}_{\left(\mathrm{ads}\right)}+{2e}^{-}+{2H}^{+}\to 2\mathrm{RSH}+\mathrm{Hg}$$

**Figure 1:**
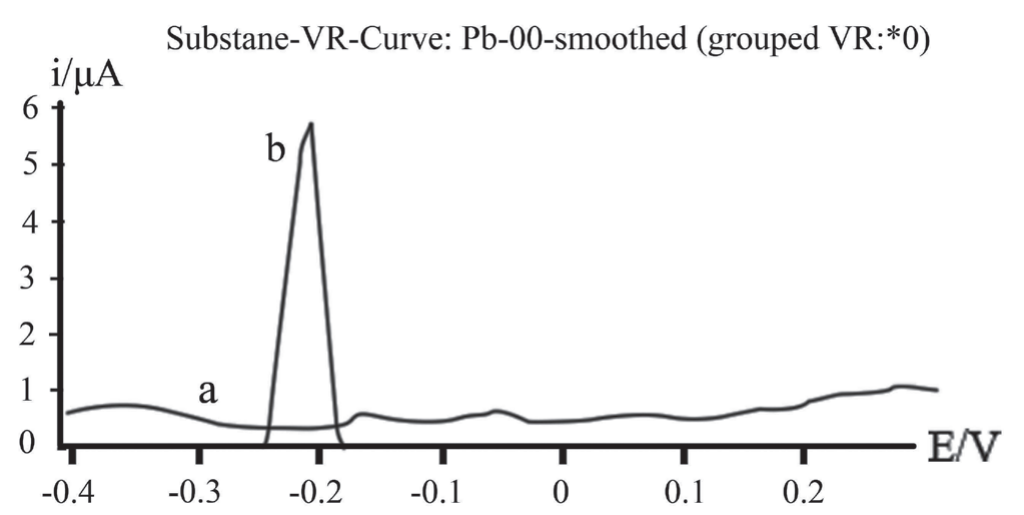
SW- AdAs voltammogram for 6 × 10^-7^ M thioctic acid in Britton Robinson buffer of pH 3.29; ƒ=60 Hz; ΔE_s_=10 mV, E_sw_=20 mV. (a), Without preconcentration; (b), Following preconcentration for 60 s at -0.4V.

The produced mercaptan gives a well – defined anodic peak at a potential of – 0.21 V vs. Ag/AgCl as show in Fig. 1. This anodic peak results from the electrochemical oxidation of mercaptan to produce disulfide by the transfer of two electrons as shown by Eq. (c):

(c) $$2\mathrm{RSH}+\mathrm{Hg}\to \mathrm{Hg}{\left(\mathrm{RS}\right)}_{2}+{2H}^{+}+{2e}^{-}$$

### Effect of type and pH of the supporting electrolyte

**Figure 2:**
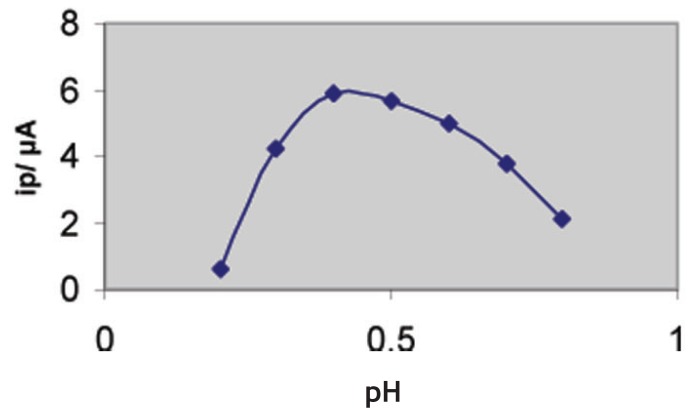
Influence of pH (Britton-Robinson buffers) on the SW – Ad AS voltammetric peak current (i_p_) of 6 × 10^-7^ M thioctic acid, ƒ=60 Hz; Δ E_s_ = 10 mV; E_sw_=20 mV; t_acc_=60 s and E_acc_=-0.4 V.

The influence of pH on the square – wave voltammetric response for 6 × 10^-7^ M thioctic acid was examined in Britton – Robinson buffers of different pH values and following pre-concentration for 60 s. The voltammograms exhibited a single well – defined two – electrons reversible anodic peak over the pH range 2-9. The peak potential shifted to more negative values on the increase of pH of the medium denoting that protons are involved in the electrode reaction process and that the proton - transfer reaction precedes the electrode process proper (33). As shown in Fig. 2, the peak current intensities (i_p_) were recorded at different pH values following pre-concentration for 60s. A much higher peak current intensity was achieved in Britton - Robinson buffers of pH 3-4. Other supporting electrolytes such as acetate buffer (pH 3.6–5.6), sodium sulphate, sodium nitrate and potassium chlolride were also tested but the peak current intensity was less developed compared to that obtained in Britton - Robinson buffers of pH3-4. Therefore Britton - Robinson buffer of pH 3.29 was used as a supporting electrolyte in the rest of present work.

### Optimization of the proposed analytical procedure

The square – wave adsorptive stripping voltammetry response markedly depends on the parameters of the excitement signal. In order to reach a maximum developed SW-Ad ASV peak current, the optimum instrumental conditions (frequency ƒ, scan increment ΔE_s_ and pulse amplitude E_sw_) were studied for 6 × 10^-7^ M thioctic acid in Britton - Robinson buffer of pH 3.29 following preconcentration for 60s. At a scan increment of 10 mV and a pulse amplitude of 20 mV, the peak current intensity increased linearly over the frequency range 20 – 60 Hz following the relationship : i_p_ (μA)=0.495 ƒ (Hz) – 2.9 (r=0.9996 and n=5). At a frequency of 60 Hz and a pulse – amplitude of 20 mV, the peak current intensity increased linearly with the scan increment up to 12 mV, following the relationship: i_p_ (μA) = 1.477 ΔE_s_ (mV) – 2.77 (r =0.9998 and n=5). Also, at ƒ=60 Hz and ΔE_s_=10 mV, the peak current increased linearly with the increase of the pulse – amplitude from 5 to 25 mV, however the best peak morphology and sharper one was obtained at 20 mV. Therfore, the optimal instrumental operational conditions of the proposed square – wave procedure can be concluded as: frequency ƒ = 60 Hz, scan increment ΔE_s_=10 mV and pulse amplitude E_sw_=20 mV.

**Figure 3:**
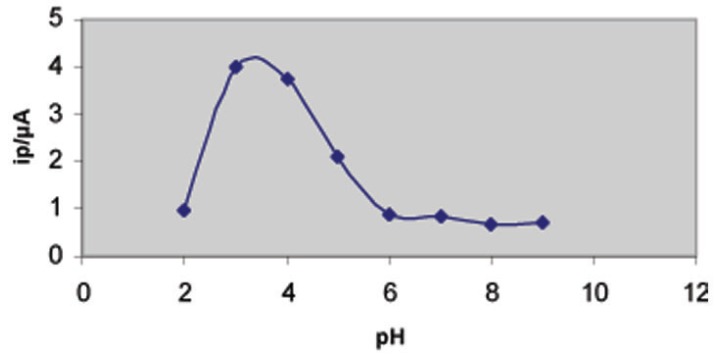
Effect of accumulation potential on the SW – AdAS voltammetric peak current (i_p_) of 6 × 10^-7^ M thioctic acid in Britton - Robinson buffer of pH3.29; ƒ=60 Hz ; E_s_=10 mV, E_sw_=20 mV and t_acc_=60 s.

On the other side, the effect of varying accumulation potential (E_acc_) from – 0.1 to – 0.6 V on the peak current intensity of the SW – Ad AS voltammogram of 6 × 10^-7^ M thioctic acid in Britton - Robinson buffer of pH 3.29 following preconcentration for 60 s was also evaluated (Fig. 3) A maximum developed peak current was achieved at the potential – 0.4 V. The observed gradual decrease in peak current intensity may be attributed to the consequence of desorption of the drug at higher or lower potential values. Hence, a preconcentration potential of – 0.4 V vs. Ag/AgCl was chosen throughout the present study.

The effect of accumulation time parameter was studied for 6 × 10^-7^ M at E_acc_ = -0.4V preconcentration potential initiated a remarkable enhancement for the SW – Ad ASV peak current up to 6os accumulation time and then it becomes nearly constant up to 150s. However at higher accumulation time, 300s, there is a decrease in peak current intensity. This may be due to desorption phenomena. Thus, for further SW-Ad ASV quantitative studies for thioctic acid, an accumulation time of 60s was selected as optimal value since it provided relatively high current with adequate practical time.

### Analytical performance of the developed procedure

**Calibration graph and detection limit.** Under the optimum experimental conditions a good linear correlation was obtained between thioctic acid electrochemical response and its concentration in the range 5 × 10^-8^ - 9 × 10^-7^ M. The parameters of the concentration – current straight line were calculated by the least squares method. The standard curve was linear with correlation coefficient (r) not less than 0.9997. The regression analysis gave the following equation:

$i_{p}\left(\mu A\right)=0.724\times 10^{7}C+1.625\;\left(r=0.9997\right)$

Validation of the method was evaluated by statistical analysis of the regression line regarding standard deviation of the intercept (S_a_=0.029) and the slope (S_b_=0.006). The small values given point to the low scattering of the points around the calibration curve.

The limit of detection (LOD) was calculated using the relation 3 (S_a_/ b) (34) and was found to be 1.2 × 10^-8^ M where S_a_ is the standard deviation of the intercept and b is the slope of the calibration curve.

**Accuracy, precision and selectivity.** The accuracy of the proposed method was checked by calculating the recovery of known amounts of thioctic acid added to Britton - Robinson buffer solution of pH3.29 and analysed via the proposed method. A mean recovery of 100.7 ± 1.23 was achieved as shown in Table 1.

**Table 1:** SW- AdASV determination of thioctic acid and its dosage forms

Drug form	% Found^a^	
	Proposed method	Reference method (5)

Thioctic acid (pure sample)	102.5	101.5
	101.0	101.1
	99.4	99.8
	101.0	
	99.6	
Mean ± S.D.	100.7 ± 1.23	100.8 ± 0.89
Student’s t-value	0.132 (2.447)^c^	
Variance ratio F-test	1.91 (19.25)^d^	
Thiotacid tablet^b^	98.0	99.1
(300 mg/tablet)	99.0	98.6
	98.5	98.3
Mean ± S. D.	98.5 ± 0.50	98.7 ± 0.40
Student’s t-value	0.543 (2.776)^c^	
Variance ratio F-test	1.56 (119)^d^	
Thiotacid tablet^b^	99.8	99.7
(600 mg/tablet)	98.8	98.6
	98.5	99.2
Mean ± S. D.	99.0 ± 0. 68	99.2 ± 0.55
Student’s t-value	0.398 (2.776)^c^	
Variance ratio F-test	1.53 (19)^d^	

a Each result is the average of three separate determinations;

b Products of Eva Pharma for Pharmaceuticals & Medical Appliance, Egypt;

c Tabulated t-values (at P=0.05) (34);

d Tabulated F–values (at P=0.05) (34).

The analytical precision of the developed method was verified from the reproducibility of 10 determinations of 3 × 10^-7^ M thioctic acid and the estimated relative standard deviation was 1.05%.

The selectivity of the optimized procedure for assay of thioctic acid was examined in the presence of some common excipients usually present in formulations e.g. starch, lactose, talc and magnesium stearate. There is no significant effect on the SW-AdASV response of thioctic acid. Accordingly, the proposed procedure can be considered selective.

### Analytical applications

The proposed method was further applied to the determination of thoictic acid in its dosage forms. As can be seen from Table 1, the analytical results achieved by the proposed SW- Ad ASV procedure were in good agreement with those obtained using the comparison method (5).

Statistical analysis of the results obtained using the student’s–test and variance ratio F–test revealed no significant difference between the performance of the two methods regarding the accuracy and precision respectively (Table 1).

## CONCLUSION

The present work describes a validated SW-AdASV method for the determination of thioctic acid without interference from common excipients. Hence, it could be applied for the routine quality control of the studied drug either in bulk or in its corresponding dosage forms. The methodology appears to be more sensitive than the reported adsorptive cathodic stripping voltammetry.
